# Laser‐induced breakdown spectroscopy/laser ablation coupled to inductively coupled plasma mass spectrometry (LIBS/LA‐ICPMS) for the forensic screening and discrimination of lead‐free solders

**DOI:** 10.1111/1556-4029.70022

**Published:** 2025-03-18

**Authors:** Kate Moghadam, Diane Beauchemin, Claude Dalpé

**Affiliations:** ^1^ Department of Chemistry Queen's University Kingston Ontario Canada; ^2^ National Forensic Laboratory Services Royal Canadian Mounted Police Ottawa Ontario Canada

**Keywords:** chemical characterization, discrimination, lead‐free solders, principal component analysis, quantitative trace analysis, tandem LIBS/LA‐ICPMS

## Abstract

The tandem LIBS/LA‐ICPMS technique is a desirable tool for the multi‐elemental determination, characterization, and classification of alloys as forensic evidence. In this study, LIBS/LA‐ICPMS is validated for the forensic evaluation of lead‐free solder alloys, which form valuable evidence from post‐blast crime scenes involving homemade and improvised explosive devices. LIBS/LA‐ICPMS is competitive with other spectroscopic‐based forensic techniques as it is in situ, analyzes samples directly, and requires minimal destruction of the exhibit. Following a one‐standard calibration technique, nine major (alloying metals) and trace elements (impurities or additives) are quantified in lead‐free solders. Optimizing laser parameters and using Pb as a naturally occurring internal standard are shown to compensate for mass‐dependent drift and matrix effects. The quantitative results of Pb‐free certified reference materials align with certificate values and with results from two techniques in a cross‐validation comparison, including electrothermal vaporization‐inductively coupled plasma optical emission spectrometry and neutron activation analysis. Utilizing peak ratios in a model of principal component analysis is presented to identify key compositional differences among solders and provide a visual model for solder discrimination. Outcomes of this approach demonstrate the potential for associating or discriminating lead‐free solders, including different solders from the same manufacturer. Together, this technique can establish chemical concordance among known and questioned materials and offers a utilitarian approach for the forensic assessment of trace evidence.

**Highlights**

- A LIBS/LA‐ICPMS method is optimized for the determination of trace elements in lead‐free solders.
- Accurate determination of elements is achieved in situ using a one‐standard calibration technique.
- LIBS alone provides fast screening of solders to differentiate lead–tin from lead‐free alloys.
- A PCA model utilizes compositional variations to associate or discriminate lead‐free solders.

## 1 INTRODUCTION

Solders from electrical circuits have been explored as trace physical evidence for the forensic exploitation of improvised explosive devices (IEDs) used in criminal acts. Following the detonation of an IED, electrical components containing solder may be recovered and chemically associated with source materials or tools used in the construction of the IED. This potential for comparison stems from variations in trace impurities and additives among different solder alloys owing to common manufacturing practices, including the provenance of raw materials (recycling, smelting processes from mining) and preferences to modify alloy properties. Several studies have demonstrated the discrimination of lead–tin solders based on their intrinsic trace element profile [1, 2, 3].

Within the last 20 years, environmental regulations (notably, the Restriction of Hazardous Substances or RoHS) have restricted the use of lead in electrical and electronic products [4]. This has driven the development of lead‐free solder alternatives such as Sn‐Ag‐Cu or Sn‐Cu alloys that contain mere traces of lead (<0.1% by weight) and greater proportions of additives. In both industrial and forensic applications, common analytical techniques for solders and related lead‐containing alloys include inductively coupled plasma mass spectrometry (ICPMS) [2, 5] and inductively coupled plasma optical emission spectrometry (ICPOES) [1, 6, 7, 8]. While these techniques are valued for their high sensitivity, they require lengthy and complex digestions that consume high masses of solder (ranging from 10 mg [1, 2] to 1 g [8, 9] per digestion). Faster and direct sampling can be achieved via X‐ray fluorescence, although this technique is usually reserved for screening purposes as a result of complications with accuracy and precision when analyzing thin or small samples [10, 11].

Laser‐based applications such as laser ablation (LA) and laser‐induced breakdown spectroscopy (LIBS) have emerged as powerful techniques for the evaluation of trace evidence. Both techniques provide unique capabilities for chemical characterization and require minimal destruction of a sample (picograms to nanograms per laser pulse) as well as the ability for in situ analysis, e.g., localized to the solder joint. LA‐ICPMS is additionally valued for its high reproducibility, sensitivity, and selectivity and relatively low limits of detection. However, quantitative analysis using LA‐ICPMS and LIBS is notoriously challenging due to laser‐induced elemental fractionation and matrix effects. Internal standards that may be easily and routinely added in solution‐based sampling cannot easily be employed with solid samples, which additionally lack appropriate matrix‐matched certified reference materials (CRMs). As a result, most LIBS applications of solder determinations are qualitative (i.e., may discriminate among lead‐rich and lead‐free solders) or are limited to the determination of Pb or Sn only using a series of CRMs [12, 13, 14, 15]. Current LIBS and LA‐ICPMS methods to quantify trace elements in solder require either a separate analytical method with acid dissolution to define an internal calibrating element [14] or the mixing of liquid standard solutions with the ablated particles [16].

A goal of this study was to introduce and validate a tandem LIBS/LA‐ICPMS technique for the detection of trace elements (Ag, As, Bi, Cd, Cu, In, Ni, Pb, and Sb) in lead‐free solders. The LIBS/LA‐ICPMS method achieves the direct sampling of solid materials with little sample preparation and without the creation of a solid standard(s) or standard solution(s). As a result, this technique increases sample throughput, minimizes the amount of sample required, and reduces the destruction of a sample compared to a digestion procedure. This is valuable for forensic investigations where exhibits must be preserved with minimal destruction for casework and court testimony, or when evidence may only be available in exceedingly small quantities. Currently, these LIBS and LIBS/LA‐ICPMS models are being explored for the analysis of other trace evidence in forensic laboratory casework, including polymer traces of 3D‐printed firearms.

The tandem LIBS/LA‐ICPMS approach also provides access to complementary chemical information of a sample, including the emission spectra of excited species (LIBS) and the mass spectrum of ions (LA‐ICPMS). LIBS/LA‐ICPMS enables a calibration approach proposed by Longerich et al. [17], which normalizes the sensitivity to the specific mass of the sample ablated, and importantly, requires only one matrix‐matched CRM for trace element quantification.

Our study incorporates a principal component analysis (PCA) model to discriminate lead‐free solders by exploiting their chemical profiles characterized using data from LIBS/LA‐ICPMS analysis. Data processing tools such as PCA have been applied widely to help answer discrimination or association‐based challenges in forensics, including models applied to the analysis of physical evidence such as paint [18], pen inks [19, 20], propellants [21, 22], and narcotics [23, 24]. PCA enables a practical solution for revealing underlying patterns in complex matrices, like solders, and elucidating trends among multi‐dimensional LIBS/LA‐ICPMS data sets. PCA is applied in this project for several purposes: first, to identify key features of the LIBS/LA‐ICPMS data set that characterize variation among solders; and second, to apply these features toward models to visualize their association or discrimination.

## 2 MATERIALS AND METHODS

### 2.1 LIBS/LA‐ICPMS instrumentation

The tandem setup combines the J200™ L LIBS/LA instrument (Applied Spectra, Inc., West Sacramento, CA, USA) with the NexION™ 300D quadrupole‐based ICPMS instrument (PerkinElmer, Woodbridge, ON, Canada). Instruments are joined directly using a 132‐cm‐long polytetrafluoroethylene tube connecting the outlet of the sample cell/ablation chamber to a T‐connector and spray chamber at the base of the torch of the ICPMS instrument. This arrangement permits the passive detection of light emission (via LIBS) as well as the extraction and measurement of ions (via ICPMS) nearly simultaneously; the same particles that are ablated in the LIBS plasma are swept to ICPMS using an Ar carrier gas.

The J200 features a 1064‐nm Nd:YAG laser source (Q‐switched ULTRA 100, Quantel USA, Bozeman, MT, USA) that is frequency‐quintupled to emit at 213 nm using a series of harmonic crystals. The pulse duration is <7 ns. The instrument is equipped with a dual‐turret Czerny turner spectrometer and an intensified charge‐coupled device (ICCD) spectrometer (Kymera 193i; Oxford Instruments‐Andor, Concord, MA, USA), which provides spectral coverage from 190 to 900 nm at either 1200 or 2400 lines/mm resolution. LIBS parameters were optimized for the detection of Pb I 405.78 nm and are listed in Table 1. Intrinsically, those parameters establish the LIBS/LA portion of the method. Using an external energy meter (Solo2™ Laser Power & Energy Meter, Gentec Electro‐Optics, Quebec City, QC, Canada), the laser energy per pulse at maximum laser power was 2.4 mJ (100 shots, 40 μm spot size, 20 Hz repetition rate, 100 warm‐up shots, 100% maximum output). Axiom™ software (version 2.0) from Applied Spectra Inc. was used to initiate ablations and collect spectra.

**TABLE 1:** Optimized parameters for LIBS/LA.

Parameter	Optimized value
*LIBS spectrometer*	
Grating	2400 lines/mm
Central wavelength and bandpass	420 ± 25 nm
Gain	90 (arbitrary units)
*Sampling and laser conditions*	
Laser spot diameter	100 μm
Gate delay	0.1 μs
Gate width	2 μs
Laser frequency	20 Hz
Laser energy per pulse	~1.8 mJ
Laser warm‐up (shutter closed)	100 shots
Laser sampling: number of shots and pattern (shutter open)	751 shots, straight line
Stage velocity and acceleration	0.01 mm/s and 0.01 mm/s^2^
Flow rate of Ar carrier gas	0.7 L/min

The NexION™ 300D features a glass T‐connector between a cyclonic spray chamber and the ICP torch to allow for better signal stability during the solder ablation. The spectrometer was operated in standard mode (no reaction/collision gas) and utilized both scanning and peak hopping for ion detection. Monitored isotopes (listed in Table 2) were selected to cover the range of trace elements expected in lead‐free solders according to the CRMs' certificates. Fe was eliminated from the study due to Ar‐based polyatomic interferences (mostly from ^40^Ar^16^O^+^ but also ^40^Ar^14^N^+^, ^40^Ar^17^O^+^, and ^40^Ar ^18^O^+^); however, a separate method with a collision/reaction gas would likely permit its determination in some solders. Optimized parameters for ICPMS are shown in Table 2. Syngistix™ software (Version 2.5) was provided by PerkinElmer and used for peak collection.

**TABLE 2:** Optimized parameters for ICPMS.

Parameter	Optimized value
Radio frequency power	1.55 kW
Ar plasma gas flow rate	18.0 L/min
Ar auxiliary gas flow rate	1.2 L/min
Make‐up gas flow rate	0.5 L/min
Monitored isotopes	^60^Ni, ^63^Cu, ^75^As, ^107^Ag, ^111^Cd, ^113^In, ^121^Sb, ^207^Pb, ^209^Bi
Number of sweeps per reading	1
Number of readings per replicate	5600
Number of replicates	1
Total acquisition time per sample	13 min

### 2.2 Standards and samples

Table 3 summarizes the CRMs and samples used in this work. Four CRMs were obtained from ARMI‐MBH Analytical Ltd. (ARMI ǀ MBH LGC Standards, Manchester, NH, USA) for use as standards for LIBS (four‐point external calibration curve) and LA‐ICPMS (single external standard calibration). These CRMs are all tin‐based lead‐free solders, varying in Pb concentration from 0.0248% to 0.183% (m/m) (Table 4).

**TABLE 3:** Solder standards and samples used in this research.

	Manufacture label	ID reference	Manufacturer	Bulk composition^a^	Form
Standards	74X E (batch F)	74X E	ARMI ǀ MBH Analytical Ltd.	^96^Sn‐^3^Cu‐^0.7^Ag	Chippings
	74X CA3 C	74X CA	ARMI ǀ MBH Analytical Ltd.	^97^Sn‐^3^Ag	Chippings
	74X HA (batch G)	74X HA	ARMI ǀ MBH Analytical Ltd.	^92^Sn‐^3^Ag‐^3^Zn‐^2^Sb‐^0.6^Cu	Chippings
	74X TC (batch F)	74X TC	ARMI ǀ MBH Analytical Ltd.	^94^Sn‐^5^Cu	Chippings
Samples	Metal Work Solder #335191	H1	Harris Products Group	Sn‐base	Spool wire
	Electrical Solder #327793	H2	Harris Products Group	Sn‐base	Spool wire
	Plumbing Solder #327790	H3	Harris Products Group	Sn‐base	Spool wire
	EDM‐4	EDM‐4	ColdHeat	Sn‐base	Spool wire
	EDM‐5	EDM‐5	Bernzomatic	Sn‐base	Spool wire
	CRM 1131	1131	NIST	40Sn‐60Pb	Disc
	91X S10PR1 (batch C)	91X S10	ARMI ǀ MBH Analytical Ltd.	9Sn‐91Pb	Disc

^a^ % (m/m) values rounded to their nearest integer from certificate.

**TABLE 4:** Comparison of measured concentrations in % (m/m) to certificate concentrations of elements in lead‐free CRMs.

	Ag	As	Bi	Cd	Cu	In	Ni	Sb
*74X TC*								
Certificate	0.039 ± 0.002	0.024 ± 0.003	0.106 ± 0.005	0.0150 ± 0.0004	4.99 ± 0.04	0.0215 ± 0.0014	0.0167 ± 0.0010	0.124 ± 0.002
Measured	0.0404 ± 0.0020	0.0308 ± 0.0031	0.1081 ± 0.0052	0.0169 ± 0.0011	4.88 ± 0.54	0.0307 ± 0.0013	0.0126 ± 0.0016	0.1104 ± 0.0060
*74X HA*								
Certificate	2.80 ± 0.03	0.0032 ± 0.0007	0.0639 ± 0.0013	0.0018 ± 0.0001	0.629 ± 0.007	0.0090 ± 0.0010	0.0133 ± 0.0005	2.10 ± 0.03
Measured	2.768 ± 0.068	0.001914 ± 0.00084	0.0721 ± 0.0011	0.001855 ± 0.000016	0.543 ± 0.025	0.00772 ± 0.00026	0.01067 ± 0.00064	2.301 ± 0.081
*74X E*								
Certificate	0.667 ± 0.007	0.0092 ± 0.0009	0.0099 ± 0.0008	0.0003 ± 0.0001	2.94 ± 0.03	0.0074 ± 0.0008	0.0069 ± 0.0004	0.0168 ± 0.0009
Measured	0.91 ± 0.16	0.0134 ± 0.0033	0.00923 ± 0.00013	0.00048 ± 0.00022	3.75 ± 0.73	0.00561 ± 0.00065	0.0086 ± 0.0015	0.0167 ± 0.0026

*Note*: Measured concentrations are shown as an average of measurements (*n* = 3, ±1 standard deviation) from three experiments performed on different days. Certificate values are presented with estimated uncertainties.

Five lead‐free solders were obtained as samples from various commercial manufacturers. Solders from Harris (Harris Products Group, Mason, OH, USA) are all silver‐bearing and contain either an acid core (Metal Work Solder, referred to as “H1”), a rosin core (Electrical Solder, “H2”), or no core (Plumbing Solder, “H3”). Both solders from ColdHeat (labeled as EDM‐4; Seattle, WA, USA) and Bernzomatic (labeled as EDM‐5; Worthington, OH, USA) contain a rosin core. Two additional reference solders, both lead‐rich, were obtained from the National Institute of Standards and Technology (NIST CRM 1131; Gaithersburg, MD, USA) and from ARMI | MBH Analytical Ltd. (91X S10).

### 2.3 Sample preparation

As solder standards from ARMI ǀ MBH were available only as thin chippings, sampling as‐is would result in ablating through the material easily. To create standards that could be regularly sampled without this risk, small aliquots of chippings (~0.4–0.5 g) were melted in ceramic crucibles using a handheld butane torch. Melting produced spherical beads that were then flattened to produce thick standard pucks. Similarly, for samples, small fragments from spool wires or discs were melted into beads and flattened. Only solder 74X HA required the addition of soldering paste flux (Kester Solder Company of Canada, Brantford, Ontario, Canada) to melt, but this did not seem to affect its use as a standard given the observed linearity of the calibration curve when 74X HA was included as a standard. Melting solder samples was done for two ends: (1) to ensure that samples would be evenly distributed with rosin (which would otherwise bias measurement should the laser spot size be focused on the rosin core) and (2) produce solder samples that would more closely resemble the evidence acquired in casework, as solders from IEDs will always be melted. The authors do not believe the butane torch had any detrimental effect on the composition or purity of the samples.

Before ablating, solder CRMs and samples were cleaned using ethanol‐soaked Kimwipes™ and then affixed to microscope slides using sticky‐tac and inserted into the ablation chamber. Following an ablation, solders were polished using 600‐grit sandpaper (Buehler Ltd., Lake Bluff, IL, USA) then cleaned again. This process removed previous ablation craters and any residual ablation particles fallout on the material surface to provide a flat, clean area for sampling.

### 2.4 Calibration strategy

LA analysis of solids is often complicated by multiplicative sources of bias, such as drift, matrix effects, and differences in the rate of ablation for samples versus standards [17]. Bias is worsened by laser power fluctuations and instability, or variations among laser‐surface interactions owing to different properties of the matrix [25]. As one solution, Longerich et al. [17] proposed an ablation sensitivity correction that selects one element, which commonly occurs in both the sample and the CRM, as the internal standard (IS). Element concentrations are calculated using a normalized sensitivity that is corrected for the mass of the sample ablated (Equation 1). In this approach, the IS must be either calculated for samples or known from CRM certificates for both materials in advance of ablation; for the samples, calculation of the IS can be accomplished using LIBS. This method is alternatively known as a single external standard calibration with an internal standard and is described as follows:

(1) $${\left[\text{analyte}\right]}_{\text{sample}}=\frac{{\left(\frac{{\mathrm{cps}}_{\text{analyte}}}{{\mathrm{cps}}_{207_{\mathrm{Pb}}}}\right)}_{\text{sample}}}{{\left(\frac{{\mathrm{cps}}_{\text{analyte}}}{{\mathrm{cps}}_{207_{\mathrm{Pb}}}}\right)}_{\mathrm{CRM}}}\times \frac{{\left[\mathrm{Pb}\right]}_{\text{sample}}}{{\left[\mathrm{Pb}\right]}_{\mathrm{CRM}}}\times {\left[\text{analyte}\right]}_{\mathrm{CRM}}$$

where [analyte]_sample_ is the unknown concentration of an element in a sample, [analyte]_CRM_ and [Pb]_CRM_ are known concentrations (certificate values) in the CRM, [Pb]_sample_ is the concentration of Pb in a sample pre‐determined by LIBS, and ${\left(\frac{{\mathrm{cps}}_{\text{analyte}}}{{\mathrm{cps}}_{207_{\mathrm{Pb}}}}\right)}_{\text{sample}}$ and ${\left(\frac{{\mathrm{cps}}_{\text{analyte}}}{{\mathrm{cps}}_{207_{\mathrm{Pb}}}}\right)}_{\mathrm{SRM}}$ are ratios of LA‐ICPMS peak signals (in units of counts/s) of an analyte and internal standard (chosen as ^207^Pb) in either the sample or CRM.

This calibration strategy was adapted for the determination of trace elements in solder. Pb is selected as the IS element and quantified in samples by LIBS using a 4‐point external calibration curve (using CRMs listed in Table 3) of the 405.78‐nm atomic emission line. These Pb concentrations were then applied in Equation (1) for the determination of other trace elements using 74X CA as the single‐point CRM. Thus, element determination is a two‐step process: first, determining the Pb concentrations by LIBS, and second, using these Pb concentrations with ICPMS peak count rates in Equation (1) to quantify all other trace elements. This strategy was adopted because most trace elements in solders were either too low to be detected by LIBS alone or did not produce interference‐free spectral emission lines. Should the Pb concentration of a sample exceed the linear range defined by the four‐point calibration curve, the recommended strategy is to build an external calibration with Pb‐rich CRMs (not shown). The same calibration strategy may be used with the exception that the laser energy be reduced due to ICCD detector saturation at the 405.78‐nm emission line, as observed with Pb‐rich solders.

### 2.5 Data processing

Time‐resolved spectra from LIBS and steady‐state signals from LA‐ICPMS were processed separately using ClarityNeXt™ software (Applied Spectra). First, LIBS time‐resolved spectra were accumulated by line (751 shots or spectra, per line). Next, accumulated Pb peaks were integrated using the integrated intensity with baseline correction feature, which finds the sum of intensities of a peak after subtraction of the linear baseline. Lastly, integrations were exported to Microsoft Excel to create an external calibration curve, which was used in turn to determine Pb concentrations in the samples.

**FIGURE 1:**
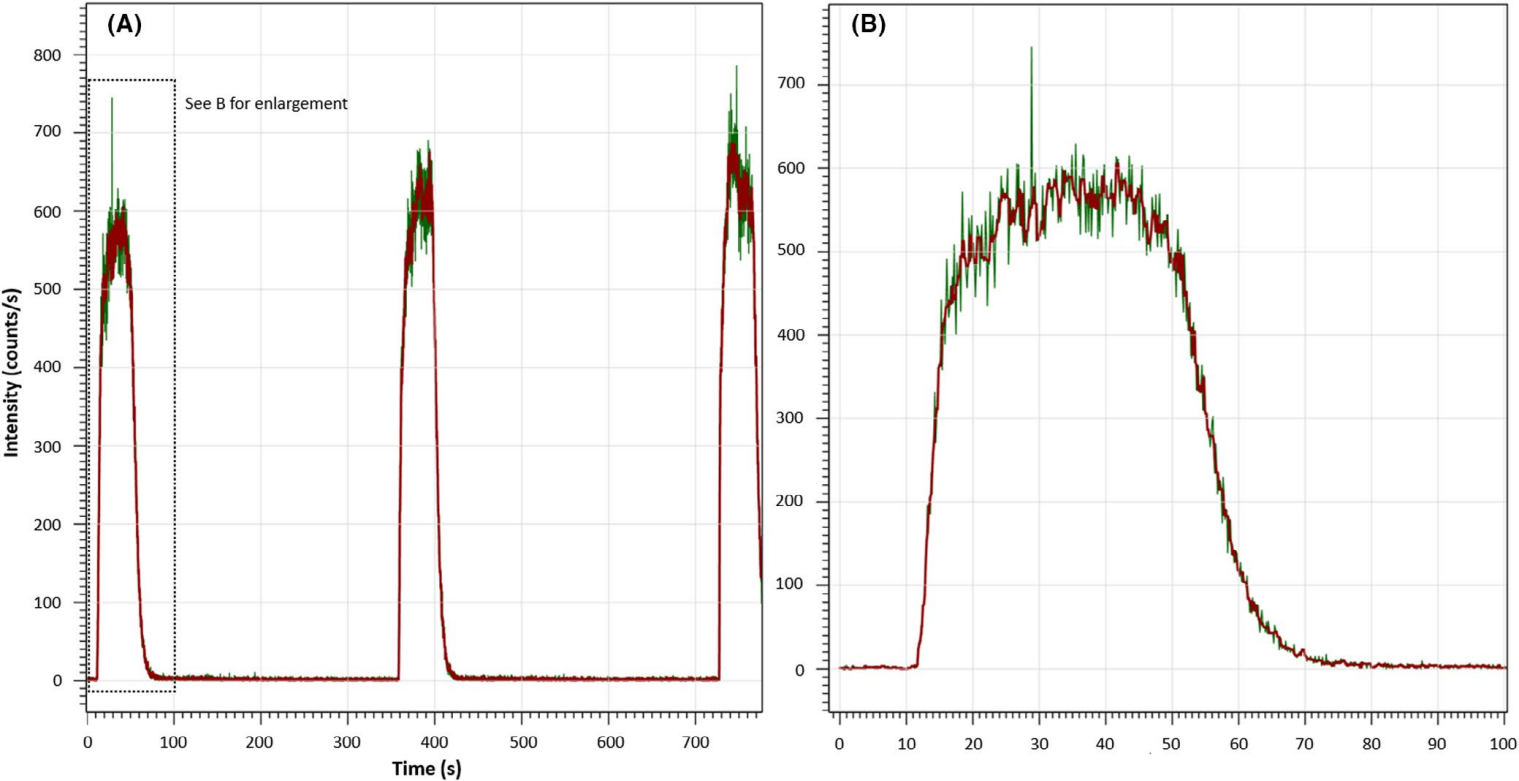
(A) Effect of median filtering on ^75^As profile from three‐line ablation of 74X E solder. The green line represents the raw signal counts without any processing. The red line presents the same data after median filtering with a window width of five points. (B) Enlargement of the first‐line ablation before and after median filtering, showing the removal of an outlier at ~28 s and smoothing of noise.

**FIGURE 2:**
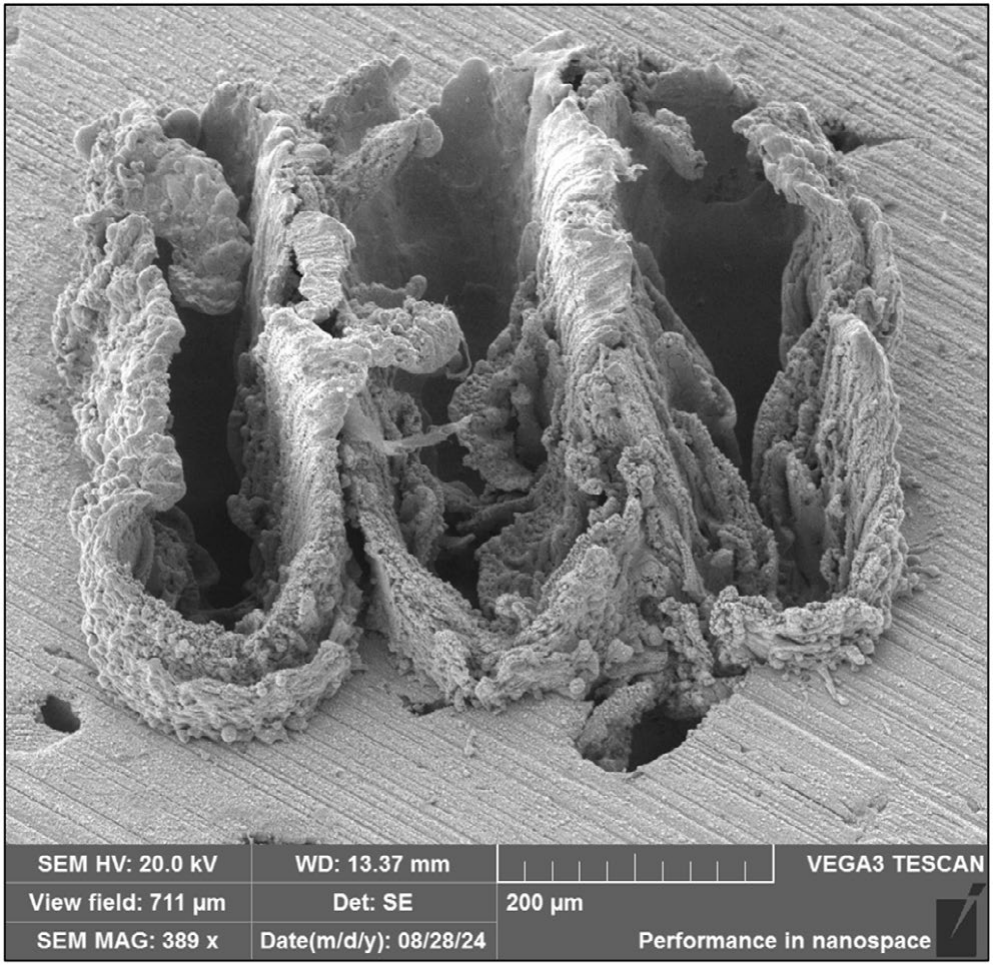
SEM image of lead‐free solder 74X CA after a three‐line ablation. Image taken using the Vega3 XMU Variable Pressure Scanning Electron Microscope (TESCAN Group, Brno, Czech Republic).

LA‐ICPMS signals were filtered by median filter smoothing with a five‐point moving window. Sinclair et al. [26] used this technique to remove extreme outliers and smooth LA‐ICPMS signal profiles, which appear noisy due to fine‐scale compositional variations in heterogeneous materials [26] or multi‐phase material with pores and voids like solders (see Figure 2). The effect of median filtering on a trace element profile is shown in Figure 1. An average of peak counts over the ablation region, minus the average of the background prior to the ablation, is used in Equation (1). This was repeated for each peak to provide an average concentration (*n* = 3) of each element.

## 3 RESULTS AND DISCUSSION

### 3.1 Optimization of LIBS/LA‐ICPMS

LIBS and LA‐ICPMS as standalone instruments require different optimization parameters than when joined in a tandem configuration. In some cases, acquisition parameters that would enhance the emission signal in LIBS might simultaneously degrade the signals observed by LA‐ICPMS, as discussed by Subedi et al. [27]. The final optimized parameters in this study reflect a compromise of conditions that provide the best analytical output from both techniques combined.

The optimization approach began first with an optimization of LIBS parameters related to the LIBS emission signal, material sampling, and the timing of data collection. When ablations were performed using a steady stream of either Ar, He, or ambient air into the ablation chamber, the highest emission signals were observed using Ar. This aligns with a report that argon produces the highest plasma temperatures and electron densities compared to helium or air [28]. A high‐resolution grating of 2400 lines/mm was used to access the highest possible resolution of the LIBS detector (~0.10 nm) and discern the low concentration Pb I 405.78 nm peak; the alternate lower resolution grating of the dual turret, 1200 lines/mm (~0.21 nm), could not effectively resolve this same peak in lead‐free solders. This high‐resolution configuration achieves a bandpass of ±23 nm from the central peak wavelength, which was selected as 420 nm to avoid peak saturation of neighboring high‐concentration analyte peaks (in particular, Sn I 380.12 nm). The gain of the LIBS detector was further increased to maximize the peak intensity of the Pb I 405.78‐nm line and improve the signal‐to‐background ratio (Table 1). A laser fluence of 45.8 J/cm^2^ (“gaussian” profile, energy output of 1.8 mJ per pulse) was found to be sufficient to observe both LIBS and LA‐ICPMS signals.

A three‐line pattern was used for ablations with a laser spot size of 100 μm. The sample stage moved at a speed of 0.1 mm/s and an acceleration of 0.1 mm/s^2^ to allow the laser to pulse a fresh sample surface each time and ensure that plasma development was not impacted by craters or residue from previous pulses [13]. A repetition rate of 20 Hz provided the most stable laser power and measurement reproducibility. Together, these parameters allowed 751 shots per line (365 μm in length and spaced 200 μm apart). Scanning electron microscopy (SEM) imaging of the solder surface following an ablation was performed and revealed that the 3‐line ablation strategy samples an area of 0.11 mm^2^, as shown by a representative SEM image in Figure 2.

**FIGURE 3:**
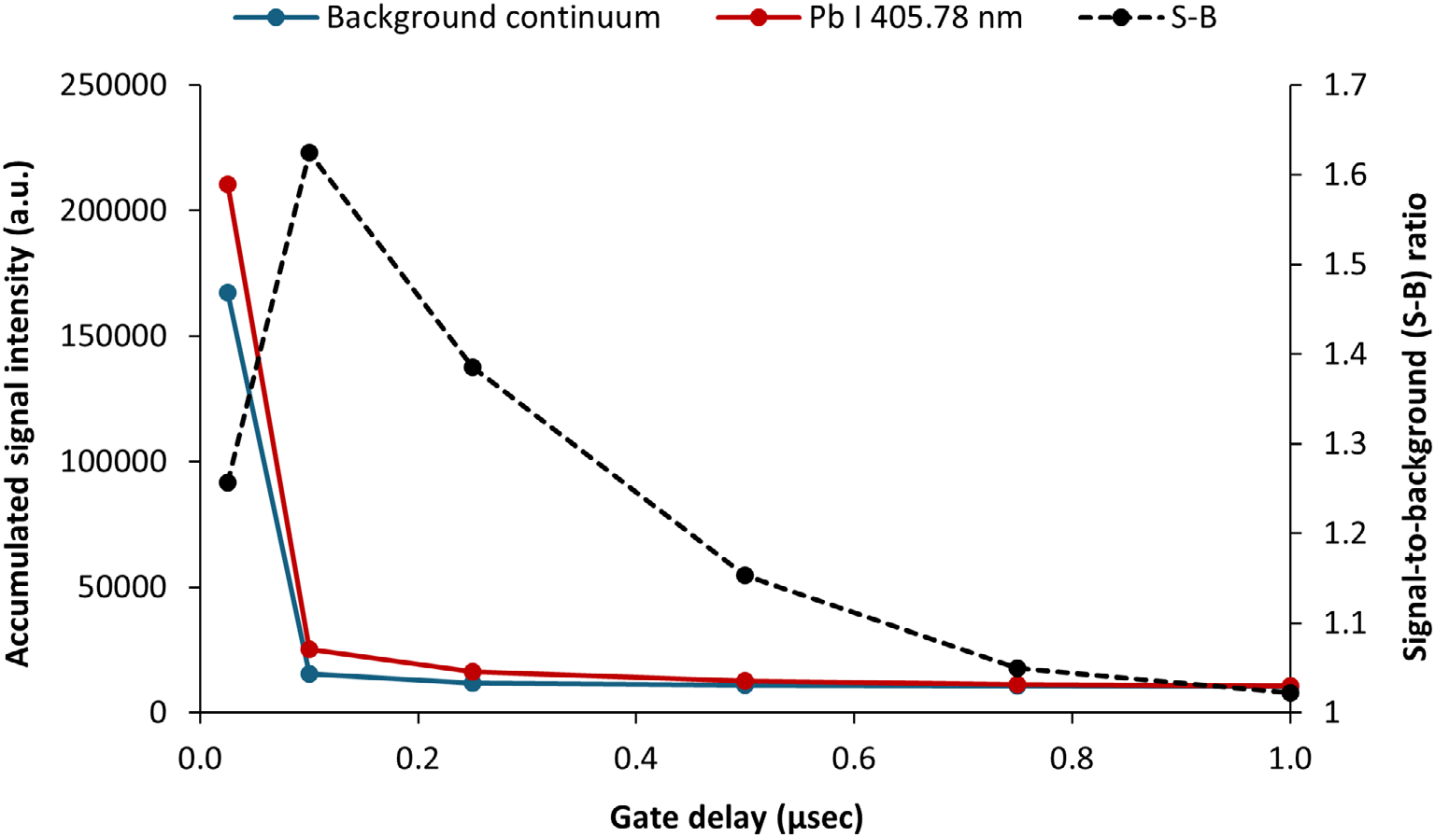
Background continuum and Pb I 405.78‐nm line emission at gate delays from 0.025 to 0.1 μs in 74X TC solder, with gate width fixed at 0.1 μs. Emission intensities are shown as accumulations from 10 laser shots.

The plasma generated by a laser pulse follows a distinct life cycle, where the emission spectra of analytes evolve with time. To determine the optimal time parameters for detection, including gate delay and gate width, the background continuum and the Pb I 405.78 nm emission line were monitored at increasing gate delays from the onset of plasma formation (when gate delay = 0 μs). The gate width remained fixed at 0.1 μs for all these measurements. As seen in Figure 3, the background continuum is most intense immediately following plasma formation. When emission is collected after a 0.1‐μs delay, the background is significantly reduced, and the signal‐to‐background ratio is greatest. Thus, a gate delay of 0.1 μs is optimal to reduce the background emission and facilitate detection of the Pb I 405.78‐nm emission line. A gate width of 2 μs was sufficient for detector measurement, as too long a gate width can introduce ambient light into the detector and degrade measurements [28].

When coupling LIBS/LA to ICPMS, key parameter adjustments included the carrier and make‐up gas flow rates. A carrier gas flow rate of 0.7 L/min was found to be optimal in maintaining a stable plasma within the sample cell/ablation chamber, while ensuring sufficient particle transport to the plasma of the mass spectrometer. An additional make‐up gas flow rate of 0.5 L/min at the T‐connector was used to dilute the aerosol particle stream to reduce plasma loading and washout time. This make‐up flow rate also ensured a steady introduction of aerosol into the torch.

Finally, efforts were made to harmonize the LIBS and LA‐ICPMS software such that ion detection would begin shortly after the laser started firing. This was done using a trigger that initiates signal acquisition in Syngistix™ once an ablation has started. Data acquisition in Syngistix™ continued for all three‐line ablations, including 5‐min washout periods between adjacent ablation lines (resulting in 5600 readings; Table 2).

### 3.2 Fast pre‐screening by LIBS

**FIGURE 4:**
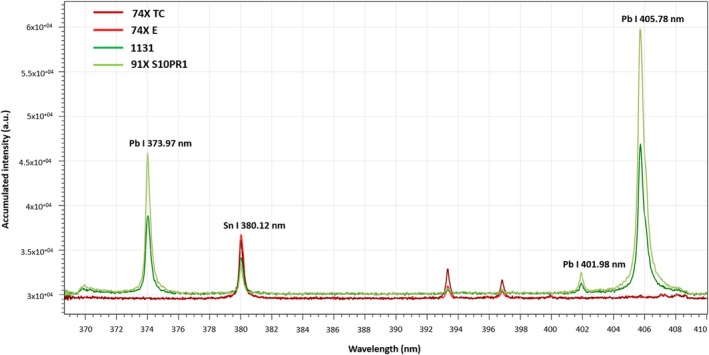
Time‐resolved emission spectra of lead‐rich solders (1131 and 91X S10, shown in green) and lead‐free solders (74X TC and 74X E, shown in red). Each spectrum is an accumulation of 61 laser shots.

LIBS alone is valuable as a pre‐screening technique to quickly distinguish lead‐free solders from lead‐rich solders. This was demonstrated using a ~10 s ablation strategy in two lead‐rich and two lead‐free solders (1 line, 61 shots, 20 Hz with 5 s warm‐up). Here, the scanning wavelength is centered at 390 nm to observe a range of both Pb and Sn emission lines, and the laser and detector conditions were adjusted to provide a laser energy of 0.6 mJ per pulse (fluence of 16.5 J/cm^2^) and a gain of 10. All other conditions followed are listed in Table 1. As shown in Figure 4, lead‐rich solders exhibit a variety of Pb atomic emission lines under these laser conditions. For lead‐free solders, the corresponding Pb peaks are not detected by the ICCD. Instead, Sn I 380.12 nm appears slightly more intense in lead‐free materials owing to the higher alloy concentration of Sn (≥94% (m/m) by weight) than in the lead‐rich solders (where Sn is 9% and 40% (m/m) by weight for 91X S10 and 1131, respectively). Two additional lines appear in the spectra of lead‐free solders, Ca II 393.38 and 396.83 nm, but these lines are not needed for alloy discrimination.

### 3.3 Performance of quantitative method

LIBS calibration curves constructed using the Pb I 405.78 nm emission line were linear and reproducible from day to day with *R* ^2^ values greater than 0.995. The limit of detection (LOD) for lead in Pb‐free solder was estimated to be 70 mg/kg (or 0.007% [m/m]), calculated as 3.3 times the standard error of the regression divided by the slope [29].

Trace element concentrations were determined in three CRMs to verify the accuracy and precision of the method using 74X CA as the external CRM and Pb concentrations from certificates. This was done day to day to provide ongoing quality control in the project. In Table 4, measured elements, shown as an average of three daily measurements, are compared to certificate values. For most elements, measured values either fall within the uncertainty interval of the certificate value or agree with certificate values according to a Student's *t*‐test (*p* < 0.05, 95% confidence interval). The difference between measured and certified values may be attributed to aliquots obtained for interlaboratory testing of a CRM, which typically involves wet chemical methods, far exceeding the aliquot masses ablated in this study. According to ARMI ǀ MBH certificates, the minimum sample size should be greater than 0.1 g, in contrast to the estimated ng‐μg quantity of material consumed by LIBS/LA‐ICPMS techniques. Despite the smaller sample size, the precision of several measured values is highly similar to corresponding certificate values, which was likely achieved by accumulating hundreds of spectra per sample and using the maximum laser spot size to offset these micro‐heterogeneities.

LODs were calculated following the procedure in Longerich et al. [17]. LODs will vary slightly for every analysis of LA‐ICPMS owing to differences in the ablated mass of each sample and further differences in the ablated mass of each *acquisition* of that sample. Although tedious, LODs should be calculated for every individual sample acquisition [17]. To provide a best approximation of LODs, values in Table 5 represent an average of LODs from three acquisitions (three lines or one ablation) of sample 74X CA.

**TABLE 5:** Estimated LODs of elements determined by LIBS/LA‐ICPMS, shown as an average from three acquisitions.

Element	LOD, ppm
Ag	0.09
As	0.06
Bi	0.06
Cd	0.2
Cu	0.2
In	0.1
Ni	0.02
Sb	0.02

### 3.4 Cross‐validation of quantitative method

The quantitative performance of the method was further evaluated by comparing the results to alternative analytical techniques of neutron activation analysis (NAA) and electrothermal vaporization coupled to ICPOES (ETV‐ICPOES). Table 6 compiles the results of this cross‐validation. Both methods involve the direct solid sampling of solder in amounts of either milligrams (ETV‐ICPOES) or grams (NAA), but only ETV‐ICPOES is destructive. Results by NAA were provided by the SLOWPOKE laboratory at École Polytechnique in Montréal, QC, Canada (D. Hall, personal communication, October 9, 2024).

**TABLE 6:** Cross‐validation of solders to various analytical techniques.

	LIBS/LA‐ICPMS (<μg)	NAA (5 g)	ETV‐ICPOES [30] (1–5 mg)
*Metal Work Solder—H1*			
Ag	0.0857 ± 0.0093	0.0743 ± 0.0039	–
As	0.0147 ± 0.0025	0.0098 ± 0.00062	–
Bi	0.00412 ± 0.00039	–	–
Cd	<DL	<0.12	–
Cu	4.0 ± 1.3	3.94 ± 0.16	–
In	0.00303 ± 0.00061	0.00292 ± 0.00012	–
Ni	0.00358 ± 0.00051	–	–
Pb	0.0237 ± 0.0023^a^	–	–
Sb	0.0082 ± 0.0014	0.00775 ± 0.00091	–
*Electrical Solder—H2*			
Ag	0.0687 ± 0.0025	0.0642 ± 0.0045	–
As	0.000996 ± 0.000017	0.00086 ± 0.00025	–
Bi	0.00193 ± 0.00013	–	–
Cd	<DL	<0.13	–
Cu	3.12 ± 0.24	3.78 ± 0.15	–
In	0.00199 ± 0.00037	0.00266 ± 0.00011	–
Ni	0.00293 ± 0.00016	<1.9	–
Pb	0.0200 ± 0.0017^a^	–	–
Sb	0.0206 ± 0.0015	0.0266 ± 0.0014	–
*Plumbing Solder—H3*			
Ag	0.092 ± 0.017	0.0702 ± 0.0037	–
As	0.00618 ± 0.00091	0.00372 ± 0.00034	–
Bi	0.0025 ± 0.0037	–	–
Cd	<DL	<0.1	–
Cu	3.86 ± 0.58	3.60 ± 0.15	–
In	0.00246 ± 0.00074	0.00346 ± 0.00014	–
Ni	0.00357 ± 0.00053	<1.7	–
Pb	0.0257 ± 0.0038^a^	–	–
Sb	0.00385 ± 0.00061	0.00322 ± 0.00074	–
*74X E*			
Ag	0.91 ± 0.16	0.602 ± 0.025	0.57 ± 0.14
As	0.0134 ± 0.0033	0.00906 ± 0.00059	0.0092 ± 0.0024
Bi	0.00923 ± 0.00013	–	0.01191 ± 0.00058
Cd	0.00048 ± 0.00022	<0.072	–
Cu	3.75 ± 0.73	2.77 ± 0.11	3.61 ± 0.42
In	0.00561 ± 0.00065	0.00861 ± 0.00035	–
Ni	0.0086 ± 0.0015	<2	–
Pb	–	–	0.0251 ± 0.0075
Sb	0.0167 ± 0.0026	0.0156 ± 0.0017	0.105 ± 0.0019

*Note*: Concentrations are reported in % (m/m) and shown with sample standard deviations (LIBS/LA‐ICPMS or ETV‐ICPOES) or uncertainties (NAA). The mass of the sample aliquot required for each method is shown in parentheses.

^a^ Concentration of Pb in H1, H2, and H3 was obtained by LIBS using the regression line of CRM peak integrations, as explained above in Section 2.5.

For all three Harris samples, results by LIBS/LA‐ICPMS compare well with NAA, with percent differences under 15% for about half of the element estimates. Expectedly, results by NAA are more precise owing to the larger sample aliquots used for measurement (5 g, corresponding to 10–40 cm of solder wire). However, the close alignment of these two techniques assures a sufficient level of accuracy in the LIBS/LA‐ICPMS method. The precision of LIBS/LA‐ICPMS is most similar to ETV‐ICPOES, as seen with 74X E estimates, where relative standard deviations (RSDs) can be as high as 20%–30%. Like ETV‐ICPOES, surface contamination and small sample aliquots have a large influence on measurement precision (potentially less representative of the overall material).

Compared with the nominal compositions provided by the manufacturer Safety Data Sheets [31, 32, 33], Cu concentrations determined by LIBS/LA‐ICPMS and NAA in H1 (4.0% and 3.94% [m/m], respectively), H2 (3.12% and 3.78% [m/m]), and H3 (3.86% and 3.60% [m/m]) are within the reported nominal range of 1 to <5% (m/m). Furthermore, the measured concentrations of Ag, when expressed to one decimal place, are in agreement with the manufacturer. For both H2 and H3, the declared nominal range of Ag is 0.1 to <1% (m/m) compared to measured concentrations of 0.0687% and 0.0642% (m/m) (for H2 by LIBS/LA‐ICPMS and NAA, respectively) and 0.092% and 0.0702% (m/m) (H3, by LIBS/LA‐ICPMS and NAA, respectively). Nominal concentrations for Ag were not indicated in the SDS of H1.

### 3.5 Solder homogeneity

**FIGURE 5:**
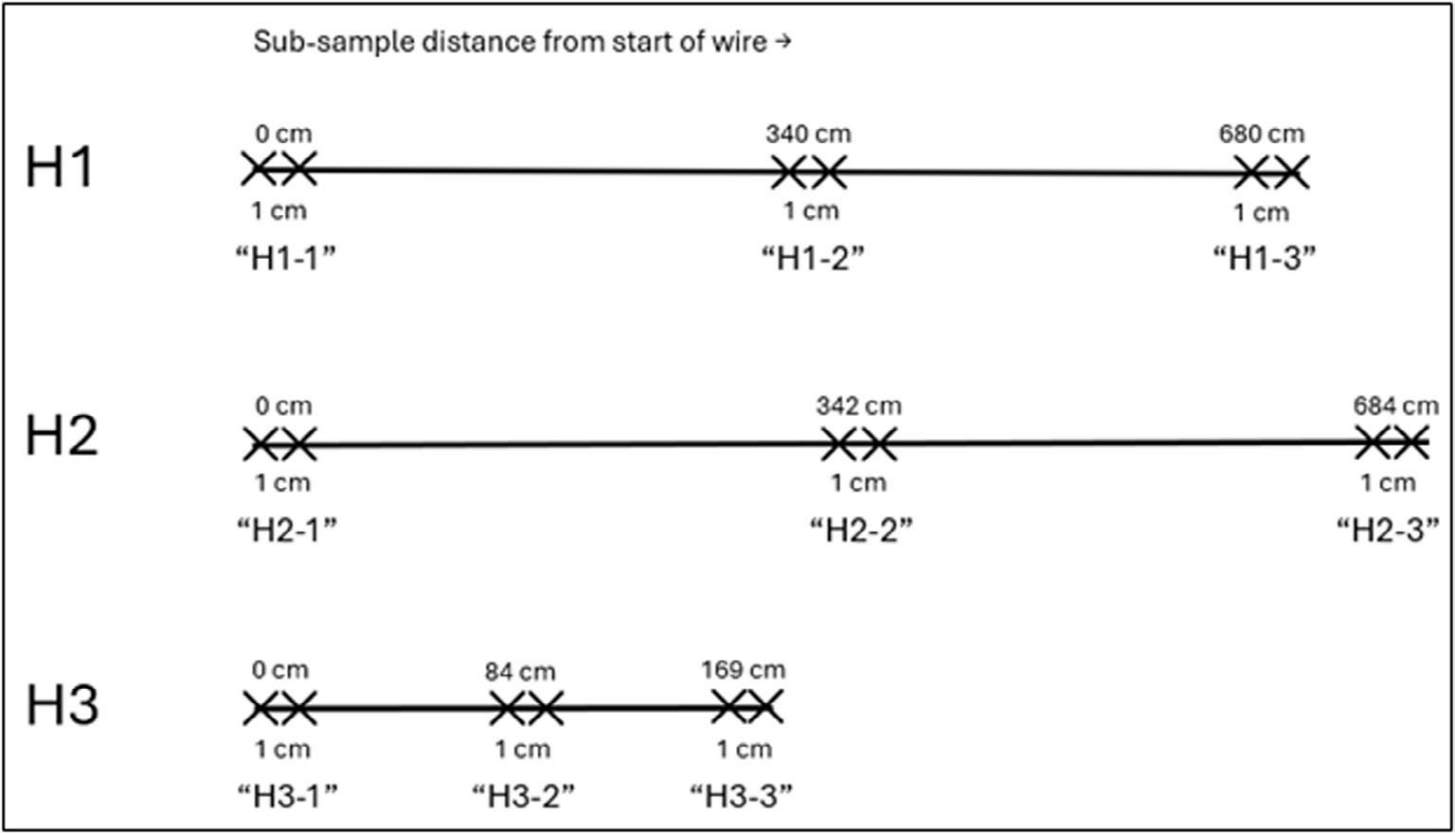
Sub‐sampling strategy of Harris solders H1–H3 depicting 1‐cm sub‐samples cut from the start, middle, and end of wires.

Trace elements are known to be slightly heterogeneous in solders, and many studies have evaluated element heterogeneity along a solder spool [1, 2, 3, 16]. A difference in concentrations among a solder spool might preclude the ability for same samples to be forensically associated, especially as smaller aliquots are obtained. In this study, element concentrations in samples H1–H3 were compared in sub‐samples collected at three locations along the entire wire length (Figure 5).

**FIGURE 6:**
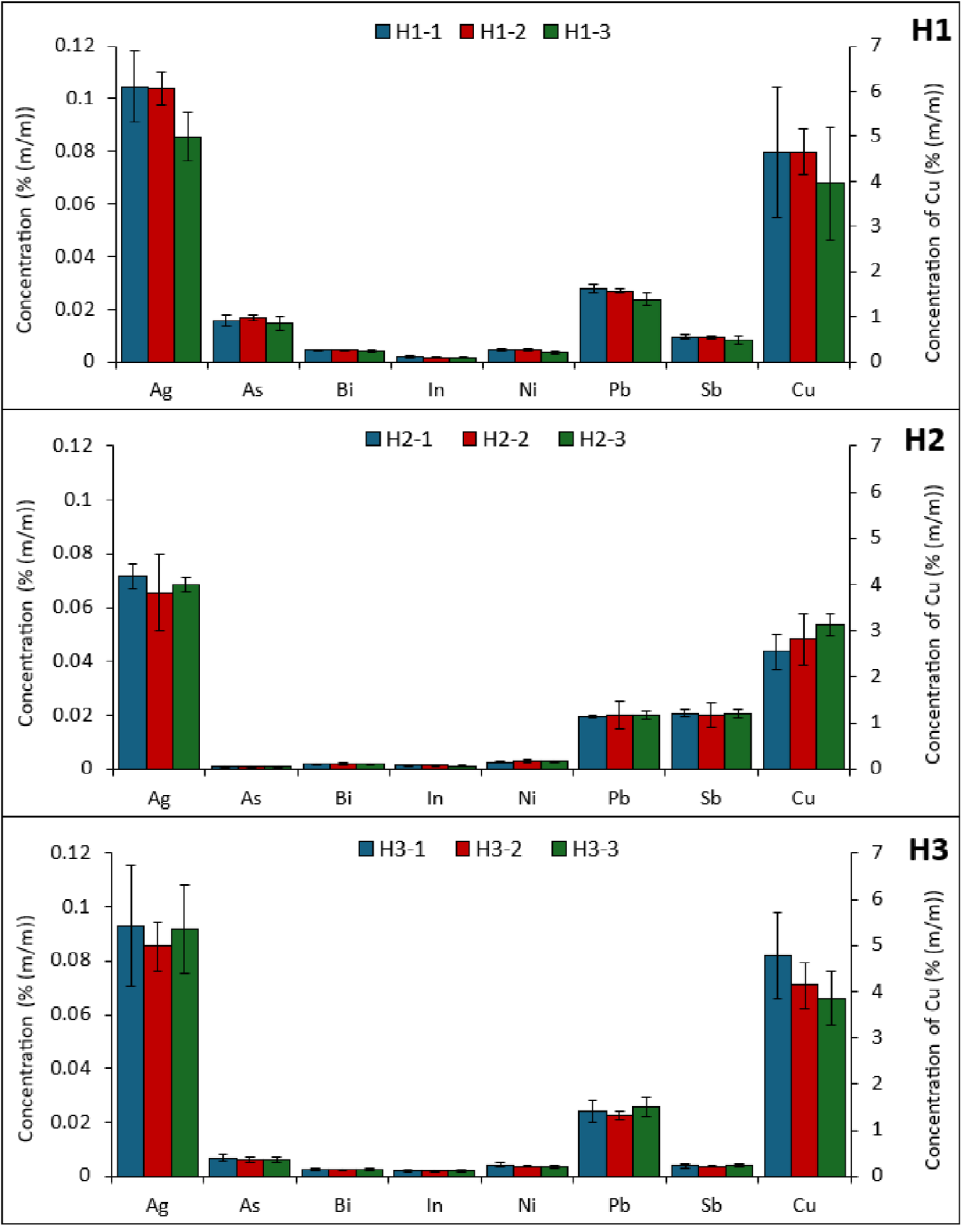
Concentrations of elements in three sub‐samples along a solder wire in H1 (top), H2 (middle), and H3 (bottom). Concentrations of Cd, not shown, are below the LOD.

Figure 6 shows the distribution of element concentrations along the solder wires. For each Harris solder, one‐way analysis of variance (ANOVA) tests were used to compare mean element concentrations among sub‐sample groups. In each case, element concentrations were found to not be significantly different among sub‐sample groups (*p* < 0.05). Similar observations were found in two solution‐based analyses of different solders using 1‐cm sub‐samples [1] or 5‐mm sub‐samples [2], including in a solid‐sampling approach using <1‐mm sub‐samples via solid‐sampling ETV‐ICPOES [3]. Therefore, minor differences in element concentrations in a solder wire should not preclude the ability for same samples to be matched or compared.

### 3.6 Principal component analysis (PCA)

PCA statistics were generated with XLSTAT™ software (Version 2023.3.1, Addinsoft©, Paris, France) using LIBS/LA‐ICPMS data from 27 measurements of lead‐free solders. Processed isotope peaks were integrated and normalized to ^207^Pb, then standardized with the z transform (or “scaling” [34]). Scaling by this method eliminates the effect of gross size and range of variation on the classification model by equalizing the influence of variables with small variation to the influence of variables with high variation. This prevents a variable with high variation from dominating the classification model because PCA is sensitive to scale differences. In a data matrix of *m* × *n*, where *m* are observations (*m* = 27, each a measurement replicate of solder) and *n* is variables (*n* = 8, peak ratios), scaling is performed across *n* rows, such that mean and sample standard deviations are calculated from ratios within an observation only. Thus, clustering can be achieved between two or more solders when their element ratios behave similarly, that is, when ratios are both either above or below their own observation mean.

**FIGURE 7:**
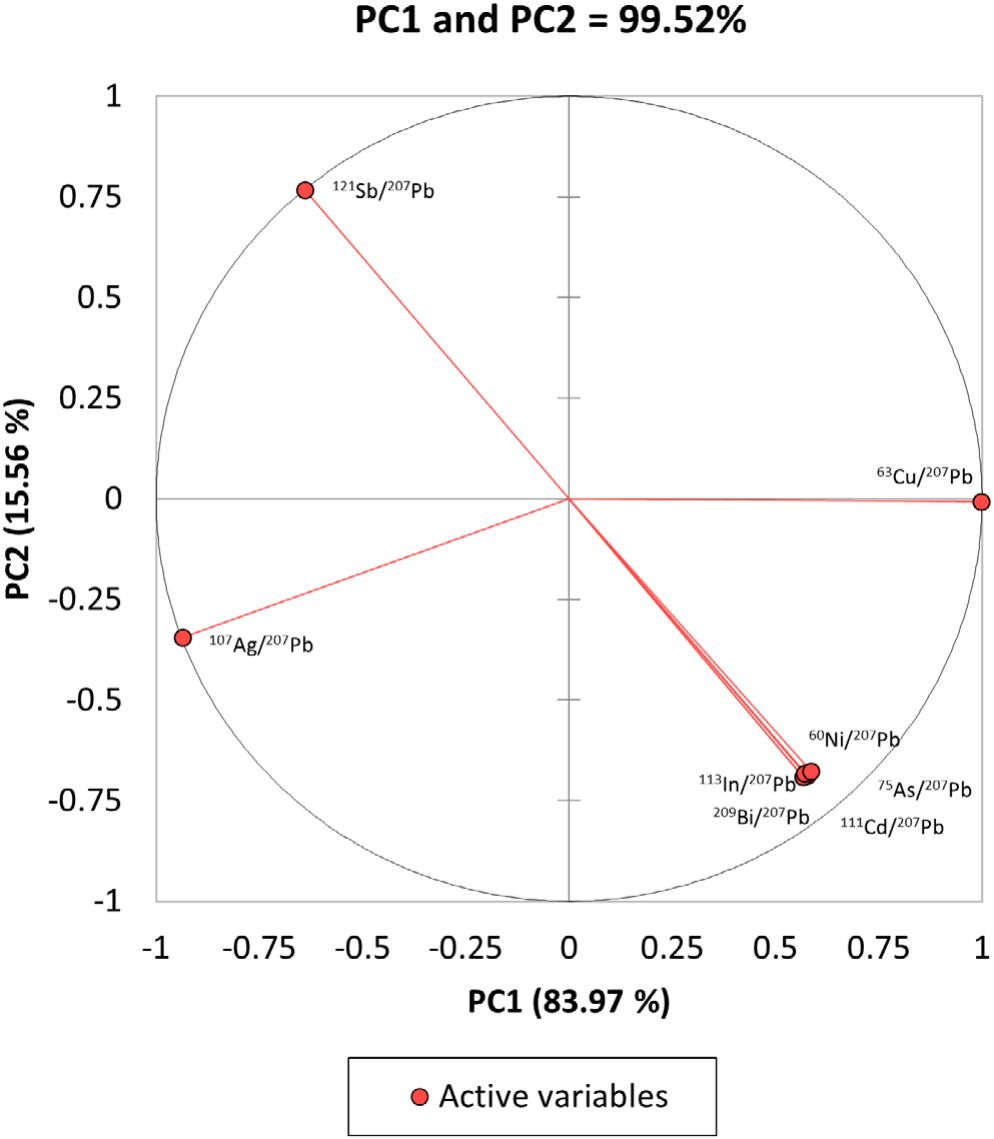
Loadings of the variables along the first two principal components PC1 and PC2 vectors that account for 99.52% of the variation. Variables well represented by the PCs have vectors that extend close to the circumference of the circle.

The first step in PCA is feature selection (variable selection). When PCA is performed using all eight peak ratio variables, the correlation circle in Figure 7 is produced. The percent contributions of features are provided in Table 7. As shown, 99.52% of the information or data variance of the original dataset is explained by the first two principal components (PCs), PC1 and PC2 linear vectors (eigenvectors). The correlation circle identifies ^63^Cu/^207^Pb as a strong contributor to PC1 but close to nil toward PC2, whereas ^107^Ag/^207^Pb and ^121^Sb/^207^Pb contribute to both PC1 and PC2. These variables are well represented by the first two PCs, as shown by the length of their vectors (positioned close to the circumference of the circle). Another observation from Figure 7 is the collinear vectors of ratios ^75^As/^207^Pb, ^209^Bi/^207^Pb, ^111^Cd/^207^Pb, and ^113^In/^207^Pb. The grouping of these variables within the correlation circle indicates they are positively correlated, meaning they behave the same for solders in this study (e.g., when ^209^Bi/^207^Pb tends to increase, ^113^In/^207^Pb will tend to increase as well). These features are less represented by the first two PCs, as indicated by their percent contributions. To reduce multicollinearity in the model, only ^75^As/^207^Pb was retained from the group.

**TABLE 7:** Percent contributions of features toward PCs.

Features	Contribution to PC1 (%)	Contribution to PC2 (%)
^75^As/^207^Pb	0.0660	0.508
^107^Ag/^207^Pb	33.1	24.1
^111^Cd/^207^Pb	0.0630	0.506
^113^In/^207^Pb	0.0630	0.506
^121^Sb/^207^Pb	9.45	73.4
^209^Bi/^207^Pb	0.0620	0.484
^63^Cu/^207^Pb	57.1	0.0180
^60^Ni/^207^Pb	0.0670	0.486

**FIGURE 8:**
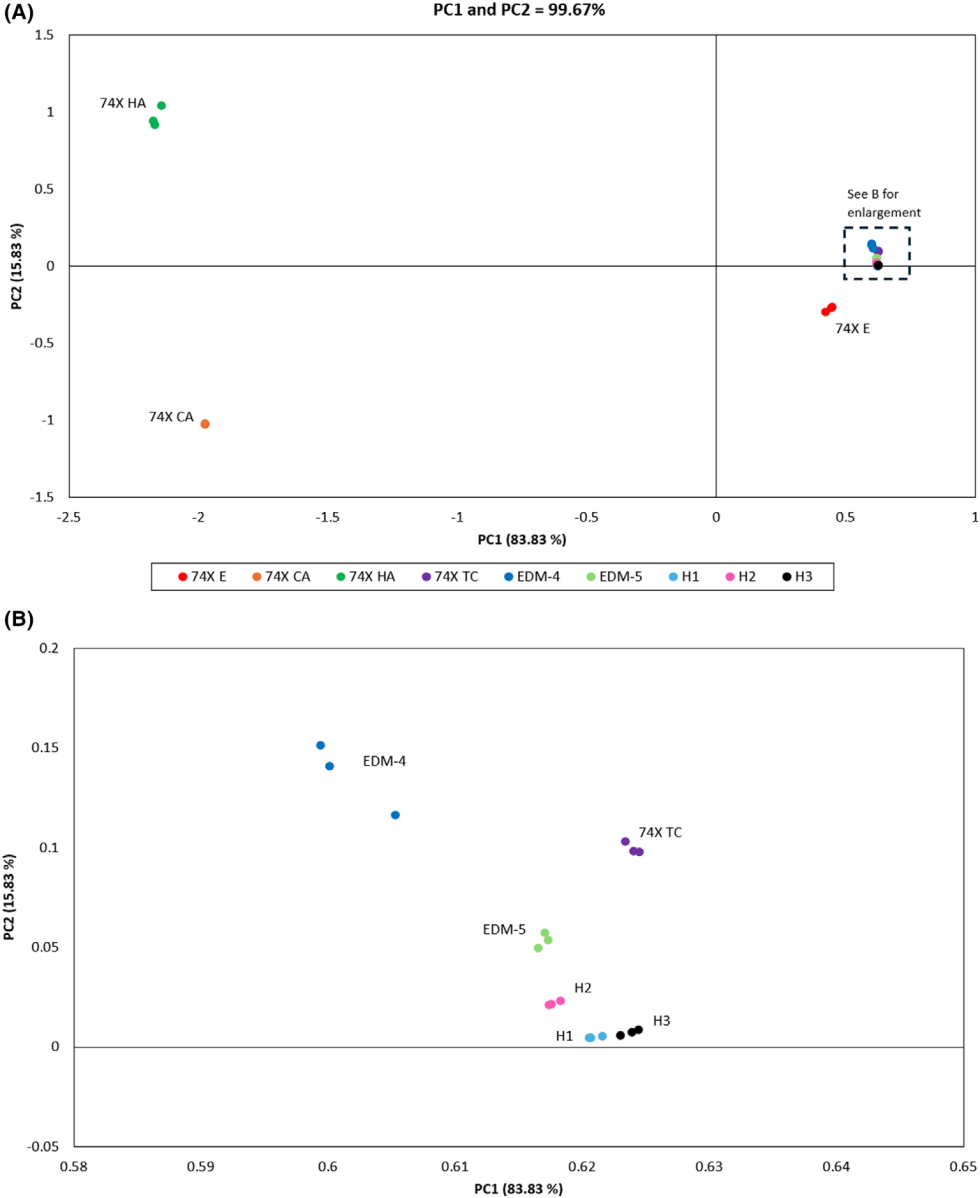
(A) 2D PCA scores plot showing the distribution and clustering of 27 measurements (scaled peak ratios) from nine lead‐free solders in the inventory. (B) An enlargement of the highlighted area from A showing the further separation of six solders.

Reducing the model to four variables (^107^Ag/^207^Pb, ^75^As/^207^Pb, ^63^Cu/^207^Pb, and ^121^Sb/^207^Pb) increased the total described variance of the first two PCs to 99.67%. Figure 8 presents the score plot of all 27 solder measurements projected along the new PC1 and PC2 eigenvectors. In this model, distinct clusters of each solder are formed. Solders 74X HA and 74X CA are separated far from the other solders due to their higher relative concentrations of Sb and Ag, and are projected in the top and bottom left quadrants following the direction of each corresponding vector in Figure 7. Intra‐class variability is very low, such that some measurements of a solder appear overlapping, as in the case for 74X CA. The inter‐class variability of the samples permits them to be clustered apart and clearly distinguished.

The two solders that are clustered close together are H1 and H3 (Figure 8B), both manufactured by Harris Group, and which contain a very similar trace element profile (recall Figure 6). Most element concentrations between the two solders were found to not be significantly different when a Student's *t*‐test (95% confidence interval) was used to compare average concentrations between sub‐samples; only elements As, Bi, and Sb were significantly different between the two solders (*p* < 0.01).

Using peak ratios rather than calculated concentrations provided by LA‐ICPMS is a faster approach for sample comparison, as it does not require an initial calibration step. The RSDs of the peak ratios range from 1% to 20% and contribute to low intra‐variability of a solder, as observed by tight clustering of samples. In cases where close clustering between two different samples occurs (H1 vs. H3), quantitative data can support the assessment of whether solders are the same or not, indicating that discrimination is possible even in the case of two lead‐free solders originating from the same manufacturer.

## 4 CONCLUSIONS

A tandem LIBS/LA‐ICPMS technique has been successfully applied for the in situ analysis of lead‐free solders. The determination of nine elements is achieved in solders without solution‐based standards, little sample preparation, and with minimal destruction to the sample. A fluence of 45.8 J/cm^2^ was suitable for the detection of LA‐ICPMS and LIBS signals and enabled low detection limits: 0.02–0.2 ppm for trace elements by LA‐ICPMS and 70 ppm for Pb by LIBS. The use of a commonly occurring element as an internal standard (Pb) and a matrix‐matched calibration material (74X CA) corrected for mass‐dependent drift and matrix effects in measurements. The results obtained for three CRMs demonstrate that the method is suitable for the quantitative detection of elements in lead‐free solders. Cross‐validation with two analytical techniques further established the accuracy of our tandem LIBS/LA‐ICPMS method.

Lead‐free solders have been found to contain different compositions of major, minor, and trace elements that can be used to differentiate one from another. A PCA model based on elemental peak ratios is demonstrated as efficient to identify these compositional variations and apply them in associating samples of the same solder, as well as discriminate different solders, even for different solder types from the same manufacturer. Our results demonstrate how qualitative peak ratios and quantitative data by LIBS/LA‐ICPMS, via one‐standard calibration, can be utilized for material characterization and discrimination. This model would support casework operations to discriminate or associate related exhibits seized from crime scenes or warrant searches, that is, match solder from a crime scene to solder seized from a suspect residence. Future work should expand the solder inventory and evaluate how other lead‐free alloys affect models of discrimination. With a greater sample set, alternative machine learning algorithms could be explored for material prediction and classification, including discriminant analysis or k‐nearest neighbors. It would also be valuable to assess solder from different manufactured batches to confirm whether source attribution can be extended to a specific production batch. Batch uniqueness would enable chemical associations to a specific lot of spools and provide additional insight into distribution or supplier sources.

Our tandem LIBS/LA‐ICPMS model may be compelling for the analysis of different types of trace evidence. Together, the instruments monitor distinct phenomena that can be either complementary or confirmatory to the identification of an analyte. Analytes that may be present in amounts too small (low ppm or sub‐ppm) to produce visible spectral peaks on LIBS may be quantifiable with the high sensitivity of ICPMS. Conversely, LIBS could quantify elements producing distinct peaks on emission spectra but which otherwise suffer from spectral interferences when determined by ICPMS. As demonstrated with our study, when LIBS is operated first, it can provide a rapid pre‐screening to identify the class or family of a material, where only 10 s was needed to determine a Pb‐free vs. a Pb‐Sn alloy base solder. Pre‐screening additionally guides the selection of an internal standard needed for quantitation, indicates matrix components in a material, and further supports the development of a suitable “menu” of isotopes to monitor by ICPMS. Finally, an area of only 0.11 mm^2^ is required for a three‐line analysis, showing our technique offers a tool for the chemical characterization of trace evidence available in only scarce quantities.

## FUNDING INFORMATION

The authors are grateful for the financial support of the Natural Sciences and Engineering Research Council of Canada (Grant No. RGPNM 39487‐2018) and of the Royal Canadian Mounted Police.

## CONFLICT OF INTEREST STATEMENT

The authors have no conflicts to declare.
